# Impact of Routine Intraoperative Lithotripsy on Surgical Strategy and Outcomes in Laparoscopic Bile Duct Exploration: A Single-Centre Cohort Study

**DOI:** 10.7759/cureus.105472

**Published:** 2026-03-18

**Authors:** Islam Elsamalouty, Aayush Gupta, Michael Whitmore, Matthew Browning, Kirk Bowling, Marcos Kostalas, Petros Christopoulos, Timothy Platt, Surajit Sinha

**Affiliations:** 1 Upper GI Surgery, Torbay and South Devon NHS Foundation Trust, Torquay, GBR; 2 Gastrointestinal Surgery, Torbay and South Devon NHS Foundation Trust, Torquay, GBR

**Keywords:** cbd stone, choledochotomy, electrohydraulic lithotripsy, laparoscopic cbd exploration, transcystic exploration

## Abstract

Background

Laparoscopic bile duct exploration (LBDE) enables single-stage management of common bile duct (CBD) stones. However, large or impacted stones often necessitate choledochotomy, which is associated with increased morbidity and longer operative duration. In 2025, routine intraoperative lithotripsy was introduced in our unit to facilitate trans-cystic stone clearance.

Aim

To evaluate the impact of routine intraoperative lithotripsy on operative approach and perioperative outcomes by comparing cases performed in 2025 with those performed between 2020 and 2024.

Methods

A retrospective cohort study was conducted of all consecutive patients undergoing LBDE between January 2020 and December 2025 at a single upper gastrointestinal surgical unit. Patients were divided into two groups: pre-lithotripsy era (2020-2024) and lithotripsy era (2025). The primary outcome was the rate of choledochotomy. Secondary outcomes included operative time, bile leak, endoscopic retrograde cholangiopancreatography (ERCP) reintervention, and length of stay. Categorical variables were compared using chi-square testing and continuous variables using Mann-Whitney U testing.

Results

A total of 301 patients were included (2020-2024: n=225; 2025: n=76). The choledochotomy rate decreased significantly following the introduction of lithotripsy (34.2% (n=77) vs 5.2% (n=4), p<0.001). Median operative time reduced from 159 minutes to 120 minutes (p=0.005). No bile leaks occurred in 2025 compared with 2.7% (n=6) in the earlier cohort (p=0.335). There were no significant differences in ERCP reintervention (7.6% (n=17) vs 5.3% (n=5.3), p=0.75) or length of stay (median 2 vs 3 days, p=0.14).

Conclusion

Routine intraoperative lithotripsy significantly reduced the need for choledochotomy and shortened operative duration. Its introduction has transformed surgical strategy toward near-universal trans-cystic completion with fewer post-operative complications.

## Introduction

Common bile duct (CBD) stones occur in around 10% of patients undergoing cholecystectomy and remain a frequent cause of biliary obstruction, pancreatitis and cholangitis. Laparoscopic bile duct exploration (LBDE) provides a single‑stage, definitive solution and avoids the need for staged endoscopic retrograde cholangiopancreatography (ERCP), reducing both the number of procedures and repeated anaesthetic exposure [1].

Two main operative routes are used to clear the duct: transcystic exploration and choledochotomy.

The transcystic route is the less invasive option and most closely mirrors a standard laparoscopic cholecystectomy, which generally means less physiological stress and a quicker recovery [2,3]. Its success, however, depends heavily on anatomy. A narrow cystic duct, high or medial insertion, or stones that lie proximally can all make this approach difficult. Large, multiple or impacted stones may also be impossible to extract through the cystic duct, pushing the surgeon towards choledochotomy instead [4]. While choledochotomy is effective, it carries recognised drawbacks, including bile leak, occasional need for T-tube drainage, longer operating times and the risk of later biliary stricture [5]. For these reasons, surgeons typically attempt a transcystic clearance first, but conversion to a choledochotomy has remained common in more complex cases.

Over the last three decades, LBDE has advanced significantly, driven by improvements in imaging, choledochoscopy and stone-extraction technology. As a result, it is increasingly viewed as the gold-standard treatment for CBD stones in suitable patients, particularly in centres with established expertise. Despite these developments, stone size and impaction remain the key factors influencing operative strategy and often necessitate a transductal approach [5].

To overcome this limitation, our unit introduced routine intraoperative bipolar electrohydraulic lithotripsy in 2025. This technique fragments large or impacted stones within the duct, potentially enabling their removal via the cystic duct and avoiding choledochotomy in cases that would previously have required it [6].

This study examines how this change in practice has influenced operative decision-making and perioperative outcomes.

## Materials and methods

This is a retrospective cohort study conducted in a single District General Hospital (DGH; Torbay and South Devon Trust Hospital) upper GI unit. The study includes all patients undergoing laparoscopic cholecystectomy with bile duct exploration between January 2020 and December 2024 for the pre-lithotripsy era and January 2025 and December 2025 for the lithotripsy era. There were no exclusion criteria.

Using cholangioscopic guidance through a transcystic approach, electrohydraulic energy is applied directly to the stone to break it into smaller, retrievable fragments. Once fragmentation is achieved, the pieces are removed endoscopically with baskets, either drawn out through the cystic duct or, when appropriate, gently pushed into the duodenum to pass spontaneously.

Effective lithotripsy depends on clear visualisation to ensure energy is delivered precisely and to minimise the risk of mucosal injury. After fragmentation and clearance, the CBD is inspected again to confirm that no residual fragments remain, as retained debris can lead to complications such as cholangitis or pancreatitis.

Patients were monitored for bile leaks during the post-operative inpatient period and for up to 14 days after discharge. Follow-up was conducted through telephone calls by our unit’s laparoscopic nurse-led follow-up service, and patients were asked to return to the hospital for surgical review if any concerning symptoms or complications were identified.

Data collection 

The data were extracted from prospectively maintained electronic spreadsheets. Variables included: bile duct approach (trans-cystic vs choledochotomy), operative duration, length of stay, bile leak and ERCP reintervention. The primary outcome examined was the rate of choledochotomy, with the secondary outcomes examined being operative duration, rate of bile leak, ERCP reintervention and the length of hospital stay.

Statistical analysis 

The chi-square test was used to assess the categorical variables, and the Mann-Whitney U test was used for continuous variables. Our significance threshold was p < 0.05. We used Python (SciPy library; https://scipy.org/) for the statistical analysis.

## Results

Patient cohort****

A total of 301 patients underwent laparoscopic bile duct exploration during the study period: 225 before the introduction of routine intraoperative lithotripsy (2020-2024) and 76 after its adoption in 2025.

Demographic differences between the two groups were modest. Patients treated in the lithotripsy era were noticeably younger than those in the earlier cohort (mean 52.1 vs 65.7 years). Body mass index was slightly higher after lithotripsy was introduced (33.0 vs 30.9 kg/m²). The sex distribution was comparable, with women forming the majority in both groups (68% vs 71%). Continuous variables were assessed with the Mann-Whitney U test and categorical variables with the χ² test. Taken together, the cohorts were broadly similar, although the age difference in particular should be borne in mind when interpreting post-operative outcomes. These data are shown in Table 1.

**Table 1:** Demographics of patients in the pre‑lithotripsy (2020–2024) and lithotripsy (2025) groups Continuous variables are presented as mean ± SD and compared using the Mann–Whitney U test (age: U=7,888, p 0.02; BMI: U=8210, p=0.27). Categorical variables were compared using the χ² test (sex distribution: χ²=0.005, p=0.944).

Variable	Pre-lithotripsy (2020–2024) n=225	Lithotripsy (2025) n=76	Statistical test	Test statistic	p-value
Age (years), mean ± SD	65.7 ± 18.0	52.1 ± 17.8	Mann–Whitney U	U=7,888	0.002
BMI (kg/m²), mean ± SD	30.9 ± 8.2	33.0 ± 6.5	Mann–Whitney U	U=8210	0.27
Male, n (%)	73 (32%)	22 (29%)	χ² test	χ²=0.005	0.944
Female, n (%)	152 (68%)	54 (71%)	χ² test	χ²=0.005	0.944

Rate of choledochotomies

Primary Outcome

The shift in practice had a marked effect on operative strategy. The rate of choledochotomy fell sharply from 34.2% (77/225) to just 5.2% (4/76), a highly significant reduction (p=0.001), which is the primary outcome of this study. This is shown in Table 2.

**Table 2:** Comparison of the rate of choledoctomy between the lithotripsy era and before its use Values are presented as the number and percentage of patients. Groups were compared using the χ² test (χ²=22.77, p < 0.001), suggesting a statistically significant reduction in the use of choledochotomies in the lithotripsy era.

	2020-2024 (pre-lithotripsy)	2025 (post-lithotripsy)
Total	225	76
Number of Choledocotomies	77	4
Percentage	34.20%	5.20%

*Secondary Outcomes* Operative efficiency improved as well. The median operating skin-to-skin time decreased from 159 to 120 minutes (mean 162 vs 141 minutes, p<0.05), as shown in Table 3. Post-operative bile leaks occurred in 2.7% (6/225) of cases before lithotripsy was introduced, but were not seen in any patients afterwards, although this difference was not statistically significant (p=0.335), as shown in Table 4. The rate of post-operative ERCP did not differ significantly following the introduction of intraoperative lithotripsy (7.6% vs 5.3%, p=0.61), as shown in Table 5. Length of stay showed a numerical reduction following the introduction of lithotripsy (mean 2.16 vs 1.65 days), but again, this did not reach statistical significance (p=0.509). Taken together, routine intraoperative lithotripsy was associated with a substantial reduction in the need for choledochotomy and shorter operating times (Table 6).

**Table 3:** Comparing the mean and median operative time between the pre and post introduction of lithotripsy Values presented as mean and median minutes. Groups were compared using the Mann–Whitney U test (U=6377.5, p < 0.05), which suggest a significant reduction in operative time post the introduction of lithotripsy.

	2020-2024 (pre-lithotripsy)	2025 (post-lithotripsy)
Mean time in minutes	162	141
Median time in minutes	159	120

**Table 4:** Comparing bile leaks pre and post the introduction of lithotripsy Values are presented as the number and percentage of patients. Groups were compared using Fisher’s exact test (p=0.335), suggesting this difference in leaks is not statistically significant.

	2020-2024 (pre-lithotripsy)	2025 (post-lithotripsy)
Total	225	76
Number of bile leaks	6	0
Percentage	2.70%	0.00%

**Table 5:** Comparing the need for ERCP pre and post the introduction of intraoperative lithotripsy Values are presented as the number of patients and percentage of total cases. Groups were compared using the χ² test (χ²=0.175, p=0.676), suggesting this was not statistically significant. ERCP: endoscopic retrograde cholangiopancreatography

	2020-2024 (pre-lithotripsy)	2025 (post-lithotripsy)
Total	225	76
Number of needed ERCPs after the operation	17	4
Percentage	7.60%	5.30%

**Table 6:** Comparing post-operative lengths of stay pre and post the introduction of lithotripsy Values presented as mean days. Groups were compared using the Mann-Whitney U test (U=3530.0, p=0.509), which is not statistically significant.

	2020-2024 (pre lithotripsy)	2025 (post lithotripsy)
Mean days stayed post operatively	2.16	1.65

## Discussion

This study demonstrates that intraoperative lithotripsy significantly altered operative strategy in LBDE. Before the availability of lithotripsy, LBDE in patients with large, multiple, or impacted stones relied mainly on conventional transcystic extraction techniques and choledochotomy-based exploration. In that era, choledochotomy rates were higher because stones that were too large, impacted, or not amenable to extraction through the cystic duct often required direct ductotomy for access and retrieval. The introduction of lithotripsy has enabled the fragmentation of difficult stones and has therefore increased the feasibility of duct clearance while reducing the need for choledochotomy in selected patients [4]. The most striking finding is the statistically significant reduction in choledochotomy from 34.2% to 5.2%, as shown in Table 2. This represents a substantial shift toward trans-cystic completion, a strategy consistently associated with reduced morbidity in the literature [2,6].

Operative duration was also significantly reduced, as shown in Table 3, likely reflecting avoidance of choledochotomy and the concomitant requirement for laparoscopic suturing and T-tube management [7,8]. Previous systematic reviews of laparoscopic lithotripsy techniques have demonstrated high stone clearance rates with acceptable safety profiles [9], supporting our observed reduction in operative complexity.

Although the bile leak rate was not statistically different, as seen in Table 3, no leaks occurred in 2025. Avoidance of choledochotomy and T-tube drainage may explain this observation, as choledochotomy and T-tube drainage have been associated with increased morbidity compared with primary closure [8]. Perhaps a larger study could increase the certainty and decrease the p-value, or dispute the finding.

ERCP reintervention and length of stay decreased numerically, as shown in Table 4 and Table 5, respectively, though these changes were not clinically significant. ERCP was performed within 30 days of surgery and was reserved for retained or suspected retained common bile duct stones, as well as post-operative biliary complications such as bile leaks. Prior comparative studies of single-stage management versus staged ERCP have demonstrated similar or improved outcomes with laparoscopic approaches [1,6], and our results align with these findings. Several systematic reviews and multicentre series have shown that, when feasible, the trans-cystic approach is associated with lower morbidity, shorter procedure time and shorter length of stay than transductal choledochotomy, while achieving comparable stone clearance for selected stones [8,10]. Meta-analyses specifically comparing transcystic (TC) and transductal (TD) LBDE report lower overall morbidity, fewer biliary complications, and shorter operative times with TC approaches [10,11]. Those pooled data emphasise that adjuncts such as choledochoscopy, baskets, balloon extraction and intracorporeal lithotripsy increase the proportion of patients amenable to TC clearance [3,11,12].

Our unit-level results mirror the effect described in the literature for centres that systematically adopt adjunctive instruments: series reporting Holmium: YAG (and other) intracorporeal lithotripsy during LBDE describe improved transcystic success in impacted or large stones that formerly required choledochotomy, with high clearance rates and acceptable complication profiles [10-12]. The systematic review of holmium laser use in LBDE summarises stone-free rates that are consistently high when laser lithotripsy is used as an adjunct to choledochoscopy, supporting the mechanistic basis for our observed reduction in choledochotomy [10].

Cochrane and other high-quality reviews addressing single-stage surgical approaches vs endoscopic staged care or T-tube drainage similarly support the safety and effectiveness of LBDE in experienced hands, and question routine T-tube use because of added morbidity and resource use [1,12-14]. Our findings of reduced choledochotomy/T-tube usage and no increase in short-term complications are concordant with those systematic conclusions.

Limitations

It is important to note that these results reflect whole-unit practice change rather than isolated lithotripsy cases. The marked reduction in choledochotomy rate demonstrates a clear strategic shift. These findings support routine lithotripsy availability in units performing LBDE, consistent with modern guideline recommendations supporting advanced laparoscopic biliary techniques [6]. 

Although the present study primarily evaluated perioperative and short-term outcomes, longer-term issues after bile duct exploration and lithotripsy may include recurrent ductal stones, biliary stricture, cholangitis, and late presentations of retained fragments. We acknowledge that a longer follow-up would be valuable to better assess these outcomes.

The study was limited to one hospital, which means that not only is the study limited in the number of different surgeons performing the operations, and so surgeons' preference in technique can't fully be excluded, but also, the number of patients included was limited, which affects the certainty of the results and is reflected in the p-value. The same ratios of different bile leakages, for example, could have had more certainty and therefore a significant p-value, had more data been utilised. Another limitation is the modest difference in patient age between groups, with the lithotripsy cohort being slightly younger, which may have influenced perioperative outcomes. A future suggestion of this trial would be to examine a similar change over multiple hospitals. This may support or dispute the argument of lithotripsy use in laparoscopic cholecystectomies.

## Conclusions

Intraoperative electromechanical and laser lithotripsy availability and use significantly reduced the need for choledochotomy and significantly and substantially reduced operative duration in LBDE at our centre. Its introduction has enabled near-universal trans-cystic completion without increasing complication rates. Lithotripsy should be considered an essential adjunct in modern LBDE practice.
